# Breast Cancer Risk Factors Analysis in Middle‐Aged Women: Evidence From a Cross‐Sectional Epidemiological Study

**DOI:** 10.1002/hsr2.72498

**Published:** 2026-08-09

**Authors:** Hourieh Ansari, Sara Yadollahi, Soheila Allahyari, Ali Hajigholami

**Affiliations:** ^1^ Family and Community Medicine Department, School of Medicine Isfahan University of Medical Sciences Isfahan Iran; ^2^ Internal Medicine Department, School of Medicine Isfahan University of Medical Science Isfahan Iran; ^3^ Internal Medicine, Division of Hematology and Oncology, School of Medicine Isfahan University of Medical Sciences Isfahan Iran

**Keywords:** breast cancer, epidemiological profile, middle‐aged women, regression analysis, risk factors

## Abstract

**Background and Aims:**

Breast cancer continues to impose a considerable burden on women's health worldwide and represents one of the primary causes of cancer‐related mortality. Despite extensive research, regional data on its distribution and associated determinants remain essential. The present study evaluated the frequency of breast cancer and explored potential related factors among middle‐aged women in Isfahan, Iran.

**Methods:**

A cross‐sectional epidemiological survey was performed between March 2021 and March 2022 across four university‐affiliated health centers in Isfahan. Information regarding sociodemographic characteristics, lifestyle behaviors, and medical history was gathered using a structured data collection form. Descriptive statistics were applied to summarize the data. Group comparisons were conducted using independent *t*‐tests for continuous variables and chi‐square tests for categorical variables. A confidence level of 95% was considered for statistical inference.

**Results:**

Among the 1001 women included in the analysis, 92 cases of breast cancer were identified, whereas 909 participants had no diagnosis of the disease. Statistical analysis revealed significant relationships between breast cancer and alcohol use (*p* = 0.014) as well as height greater than 175 cm (*p* = 0.048). No meaningful associations were observed for the remaining evaluated variables (*p* > 0.05).

**Conclusion:**

The study findings indicate a potential link between alcohol consumption, increased height, and breast cancer occurrence in middle‐aged women. Further large‐scale, multi‐center investigations are warranted to confirm these observations and to enhance understanding of modifiable and non‐modifiable risk factors that may inform preventive strategies.

## 1 Introduction

Globally, breast cancer remains the leading malignancy among women, with a substantial burden in both high‑ and low‑resource settings [1]. Although incidence rates are higher in wealthy nations, mortality rates are disproportionately elevated in less developed countries [2, 3]. In high‑income regions such as the United Kingdom, Australia, and Eastern Europe, more than 60% of cases are detected at early stages (I or II), which considerably improves prognosis [4]. By contrast, women in low‑income countries frequently present with metastatic disease at the time of diagnosis [5, 6, 7, 8]. Projections indicate that by 2040, annual new breast cancer cases will exceed 3 million (a > 40% increase), and annual deaths will reach 1 million (a > 50% increase from 685,000 in 2020) [9]. In Iran, breast cancer is also the most prevalent cancer among women [10].

Breast cancer is a multifactorial disease resulting from the complex interplay of genetic, hormonal, lifestyle, and environmental factors. These risk factors are broadly categorized as non‐modifiable and modifiable. Non‐modifiable risk factors include advancing age, female sex, a positive family history of breast cancer, inherited genetic mutations (e.g., BRCA1/2), and a personal history of certain other malignancies [11, 12, 13, 14, 15]. Reproductive factors with a significant non‐modifiable component include early age at menarche, late age at menopause, nulliparity, and older age at first birth, while a history of breastfeeding is recognized as a protective factor [11, 12, 13, 14]. Conversely, modifiable risk factors, which are amenable to intervention, encompass lifestyle and behavioral elements such as physical inactivity, postmenopausal obesity (high BMI), alcohol consumption, tobacco smoking, and inadequate intake of fruits, vegetables, and vitamin supplements [16, 17, 18]. It is noteworthy that, according to the American Cancer Society, established risk factors account for only approximately 25% of breast cancer cases, underscoring the complexity of its etiology [6].

The risk factor profile for breast cancer has been explored in various populations, including Iran, where studies have identified several key domains. Genetic and historical factors, such as aging and family history, remain prominent [19]. Hormonal and reproductive factors, including early menarche, late menopause, and specific pregnancy‐related characteristics, are also consistently reported [20, 21, 22, 23]. Furthermore, lifestyle‐related risks, notably obesity and poor dietary habits, alongside medical interventions like hormone replacement therapy (HRT) and chest radiation therapy, contribute to the overall risk [20, 21, 22, 23]. Environmental and occupational exposures have also been implicated [24, 25]. This multifaceted array of factors highlights the complexity of breast cancer risk and reinforces the critical need for targeted prevention strategies and early detection efforts, particularly in populations with unique demographic and cultural characteristics.

Several risk factors for breast cancer have been identified in prior research. Given the high incidence and prevalence of this disease, its substantial treatment costs, the vulnerability of socioeconomically productive women (aged > 35 years), and the absence of nationwide screening or early detection programs, breast cancer constitutes a major public health priority [26, 27, 28]. Despite all the efforts made by researchers and screening programs in countries, the incidence of breast cancer continues to rise. As a first step towards reducing breast cancer incidence, it is necessary to reduce exposure to modifiable risk factors, so it is crucial to identify breast cancer risk factors to develop preventive strategies. Regarding the critical role of identifying breast cancer risk factors, we conducted a cross‐sectional study on the women who were admitted to four of Isfahan University's affiliated academical health centers to better evaluate the prevalence of breast cancer risk factors in the setting of our regional health policies and environmental status.

## 2 Methods

### 2.1 Study Design

This cross‐sectional epidemiological study was conducted in four of Isfahan university affiliated academical health centers from March 2021 to March 2022 and explored prevalence of breast cancer risk factors among middle‐aged women. Four comprehensive health centers in Isfahan were selected using a simple random sampling method. From the list of middle‐aged women covered by these centers, 1001 women were systematically randomly selected and enrolled in the study based on the inclusion criteria. Then our checklist was completed by researchers through phone calls and face‐to‐face visits. The study protocol has been approved by the institutional ethics committee (IR.MUI.MED.REC.1401.186).

### 2.2 Inclusion and Exclusion Criteria

The inclusion criteria were women aged 30–59 who were covered by the health centers listed who had signed the informed consent. Those participants who did not complete more than 20 percent of the questions were excluded.

### 2.3 Sample Size

The sample size was calculated using Equation (1) and considering *α* = 0.05, *P* = 50%, *Z* = 1.96, *P* = %50 and a maximum estimation error of 0.031 [2].

(1) $$N=\frac{p\left(1-p\right)\times {z(1-\frac{\alpha }{2})}^{2}}{d^{2}}$$

Based on the above parameters, the minimum required sample size was calculated as 1067 participants. After accounting for potential incomplete responses, a target sample of 1100 individuals was set. Following data collection and cleaning, 1001 complete questionnaires were included in the final analysis. The achieved margin of error was recalculated as 0.031 (3.1%), which remains within an acceptable range for epidemiological studies. This sample size was selected using a list frame and a simple random sampling method from women aged 30–59 years covered by comprehensive health education centers in Isfahan city.

### 2.4 Data Collection

Since the samples were selected from health centers that provide routine healthcare services for middle‐aged women, it is important to clarify how participants came to undergo mammography in the absence of a national screening program. In this setting, healthcare staff routinely offered counseling sessions during women's regular visits, during which they provided motivational guidance and emphasized the importance of early breast cancer detection. These efforts were aimed at encouraging eligible women to voluntarily undergo mammography. Following these consultations, women who agreed to participate were referred for mammography, and data were subsequently collected using a 43‐item checklist developed by the research team. all the participants were asked for demographic characteristics, past medical history and reproductive factors including age, marital status, educational status, job, family history of breast cancer in first degree relatives, pregnancy history, age of first pregnancy, number of breast feeding months, age at menarche, menopausal status, menopause age, BMD Z‐score, physical activity, fruit and vegetable consumption, vitamin supplementation, history of infertility and duration and type of treatment, history of other malignancies, breast cancer in male family member, HRT, smoking, alcohol intake, exposure to radiation therapy of chest, weight, height and BMI > 30 before menopause. This checklist was based on last versions of middle‐aged guidelines (2022) and UpToDate and was approved by a nutrition specialist and oncologists.

### 2.5 Data Analysis

All statistical analyses were performed using SPSS software (version 26.0, IBM Corporation, Armonk, NY, USA). Descriptive statistics were computed for all study variables. Given that all variables in this study were categorical, they were summarized as frequencies and percentages. To examine the association between each potential predictor and breast cancer status, we conducted univariate analysis using the Pearson chi‐square test or Fisher's exact test where expected cell frequencies were less than 5. All statistical tests were two‐sided, and a *p*‐value of less than 0.05 was considered statistically significant.

## 3 Results

In this epidemiological study, a total of 1001 people enrolled in this study and completed questionnaires. The highest age frequency was observed in the 30–35 age group (27.5%), while the lowest was in the 45–50 age group (14.7%). 91.7% of the participants were married, and 75.4% were housewives. A height above 175 cm was reported in only 1.1% of individuals; however, it was identified as one of the significant risk factors in the analysis results. A BMI over 30 (obesity) was reported in 23.5% of the overall participants. Regarding reproductive history, 89.1% of the women had experienced pregnancy, and 88.3% had breastfed, both of which are important protective factors against breast cancer. The onset of menstruation at age 12 or younger was reported in 30% of participants, which may indicate a potential impact on breast cancer risk (Table 1).

**Table 1:** Demographic information and distribution of women undergoing breast cancer screening (*N* = 1001).

Factor	Variable	Category	Percent (%)
Demographic factors	Age	30–35	27.5
		35–40	16.4
		40–45	19.3
		45–50	14.7
		50–60	22.2
	Marital status	Single	6.5
		Married	91.7
		Divorced	1.8
	Job	House Keeper	75.4
		Retired	2.7
		Employed	21.9
	BMI	< 30	76.5
		≥ 30	23.5
	Height > 175 cm	Yes	1.1
		No	98.9
	Education	Below Diploma	27.4
		Diploma	32.7
		Higher	39.9
	Osteoporosis	No	8.1
		Yes	91.9
Reproductive factors	Age of first menstruation	≤ 12	30.0
		13–14	48.0
		≥ 15	22.0
	Menopausal status	Yes	22.3
		No	77.7
	Age of menopause	< 55	89.7
		≥ 55	10.3
	History of infertility	Yes	4.9
		No	95.0
	History of pregnancy	Yes	89.1
		No	10.9
	Age of first pregnancy	≤ 29	88.1
		≥ 30	11.9
	Positive breast cancer biopsy	Yes	3.5
		No	96.5
	breastfeeding	No	11.7
		Yes	88.3
Self and Familial history	Positive history of breast cancer in first‐degree relations	Yes	9.2
		No	90.8
	History of other malignancies	Yes	0.5
		No	99.5
	History of hormone therapy	Yes	1.6
		No	98.4
	History of benign breast cancer	Yes	19.6
		No	80.4
	History of benign high‐density lesions	Yes	6.7
		No	93.3
	Overweight before menopause	Yes	14.8
		No	85.2
	Overweight after menopause	Yes	8.2
		No	91.8
	History of cancer in first‐degree relatives	Yes	46.2
		No	53.8
	History of cancer in second‐degree relatives	Yes	49.9
		No	50.1
Life‐style factors	History of alcohol consumption	Yes	1.7
		No	98.3
	History of smoking	Yes	1.7
		No	98.3
	History of passive smoking	Yes	26.7
		No	73.3
	History of chest radiation	Yes	0.4
		No	99.6
	Physical activity (in the last 5 years)	Barely	47.7
		Moderate	21.9
		Good	30.3
	30 min exercise 3–5 days in week	Yes	52.0
		No	48.0
	Taking fruits weekly (in last 5 years)	Barely	14.5
		Moderate	31.4
		Good	54.1
	Taking vegetables weekly (in last 5 years)	Barely	21.6
		Moderate	36.0
		Good	42.4
	Soy consumption weekly (gr)	0	44.7
		< 50	8.6
		≥ 50	46.8
	Taking vitamin D supplements	Yes	67.0
		No	33.0
	Taking calcium supplements	Yes	23.0
		No	77.0
	Taking dairy products daily	Yes	91.5
		No	8.5
	Taking meat products daily	Yes	99.4
		No	0.6

In total, 92 patients had breast cancer, and 909 of them were non breast cancer. Among 92 individuals diagnosed with breast cancer, a height of over 175 cm was significantly more prevalent at 3.3% compared to the non‐affected group (0.9%) (*p* < 0.05). No significant differences were observed between the two groups regarding other demographic characteristics such as marital status, occupation, and BMI (Table 2).

**Table 2:** Comparison of demographic information of women undergoing breast cancer screening (negative and positive).

Factor	Category	Positive cancer (%)	Negative cancer (%)	*p*
Age	30–35	25.0	27.8	0.282
	35–40	14.1	16.6	
	40–45	15.2	19.7	
	45–50	18.5	14.3	
	50–60	27.2	21.7	
Marital status	Single	5.4	6.6	0.307
	Married	91.3	91.7	
	Divorced	3.3	1.7	
Job	House Keeper	78.3	75.1	0.483
	Retired	1.1	2.9	
	Employed	20.7	22.0	
Osteoporosis	No	10.9	7.8	0.656
	Yes	89.1	92.2	
BMI	< 30	7.8	76.8	0.890
	≥ 30	92.2	23.2	
Height > 175 cm	Yes	3.3	0.9	**0.048**
	No	96.7	99.1	

In terms of reproductive factors, early menopause (before the age of 55) was more prevalent in the affected group compared to the non‐affected group (100% vs. 88.3%, *p* > 0.05), although this difference did not reach statistical significance. Regarding lifestyle, alcohol consumption was reported at 4.3% in the breast cancer group, while it was 1.4% in the non‐affected group, which is statistically significant (*p* < 0.05). Additionally, low vegetable consumption showed a marginal association with an increased risk of breast cancer (*p* > 0.05), which may indicate the importance of a healthy diet (Tables 3 and 4).

**Table 3:** Comparison of pregnancy and lactation information of women undergoing breast cancer screening (negative and positive).

Factor	Category	Positive cancer (%)	Negative cancer (%)	*p*
Age of first menstruation	≤ 12	28.6	30.1	0.310
	13‐14	45.1	48.4	
	≥ 15	26.4	21.5	
Menopausal status	Yes	29.3	21.6	0.263
	No	70.7	78.4	
Age of menopause	< 55	100	88.3	0.098
	≥ 55	0	11.7	
History of infertility	Yes	5.4	4.8	0.920
	No	94.6	95.0	
History of pregnancy	Yes	91.3	88.9	0.644
	No	8.7	11.1	
Age of first pregnancy	≤ 29	87.1	88.2	0.278
	≥ 30	12.9	11.8	
History of other malignancies	Yes	2.2	0.3	0.150
	No	97.8	99.7	
History of positive biopsy	Yes	7.6	3.1	0.165
	No	92.4	96.9	
History of benign breast cancer	Yes	28.6	18.7	0.087
	No	71.4	81.3	
History of benign high‐density lesions	Yes	13.0	7.5	0.105
	No	87.0	92.5	
Months of breastfeeding	No	10.9	11.8	0.455
	Yes	89.1	88.2	
History of hormone therapy	Yes	1.1	1.7	0.462

**Table 4:** Comparison of lifestyle information of women undergoing breast cancer screening (negative and positive).

Factor	Category	Positive cancer (%)	Negative cancer (%)	*p*
History of alcohol consumption	Yes	4.3	1.4	**0.014**
	No	95.7	98.6	
History of smoking	Yes	1.1	1.8	0.998
	No	98.9	98.2	
History of passive smoking	Yes	30.4	26.3	0.339
	No	69.6	73.7	
History of chest radiation	Yes	1.1	0.3	0.431
	No	98.9	99.7	
Physical activity in last 5 years	rarely	47.8	47.7	0.660
	Moderate	26.1	21.5	
	Good	26.1	30.8	
30 min exercise 3–5 days in week	Yes	52.7	51.9	0.418
	No	47.3	48.1	
Taking vegetables daily	Rarely	33.3	66.7	0.019
	Moderately	26.4	73.6	
	Very often	26.4	80.6	
Taking Dairy products	Yes	90.2	9.8	0.999
	No	90.3	9.7	
Taking vitamin D	Yes	64.1	35.9	0.705
	No	66.4	33.6	
Taking Calcium supplements	Yes	23.9	76.1	0.776
	No	22.5	77.5	
Taking meat products and eggs	Yes	97.8	2.2	0.259
	No	99.3	0.7	
Soy consumption	No	39.1	42.1	0.873
	< 50	10.9	10.7	
	≥ 50	50.0	47.1	

A history of cancer in first‐degree relatives was reported in 45.1% of the affected group, which was significantly higher than in the non‐affected group (19%) (*p* < 0.001). Additionally, the odds ratio analysis indicated that alcohol consumption (OR = 11.1; 95% CI: 1.19–111.11) and a height greater than 175 centimeters (OR = 10.41; 95% CI: 1–111.11) were identified as significant risk factors. In contrast, a high intake of vegetables had a protective effect, although this effect was not statistically significant (OR = 1.84; *p* > 0.05) (Tables 5 and 6).

**Table 5:** Comparison of Self and familial history information of women undergoing breast cancer screening (negative and positive).

Factor	Category	Positive cancer (%)	Negative cancer (%)	*p*
History of pregnancy	Yes	91.3	88.9	0.644
	No	8.7	11.1	
Age of first pregnancy	≤ 29	87.1	88.2	0.278
	≥ 30	12.9	11.8	
History of other malignancies	Yes	2.2	0.3	0.150
	No	97.8	99.7	
History of positive biopsy	Yes	7.6	3.1	0.165
	No	92.4	96.9	
History of benign breast cancer	Yes	28.6	18.7	0.087
	No	71.4	81.3	
History of benign high‐density lesions	Yes	13.0	7.5	0.105
	No	87.0	92.5	
Months of breastfeeding	No	10.9	11.8	0.455
	Yes	89.1	88.2	
History of hormone therapy	Yes	1.1	1.7	0.462
	No	98.9	98.3	
History of malignancy in first‐degree relatives	Yes	45.1	19	< 0.001
	No	54.9	81	

**Table 6:** Binary logistic regression for the predictors of breast cancer patients.

Factor	Category	Univariate OR (95%CI)	Multivariable OR (95%CI)
History of alcohol consumption	Yes	3.34 (2.08–5.36)	11.1 (1.19–111.11)
	No	Ref	Ref
History of malignancy in other relatives	Yes	2.38 (1.70–3.33)	2.94 (1.69–5)
	No	Ref	Ref
Height > 175 cm	Yes	3.10 (1.71–5.62)	10.41 (1–111.11)
	No	Ref	Ref
Taking vegetables weekly (in last 5 years)	rarely	2.08 (1.4–3.90)	1.84 (0.94–3.59)
	Moderate	1.49 (0.87–2.57)	1.58 (0.88–2.82)
	Good	Ref	Ref

## 4 Discussion

This study was aimed to evaluate the prevalence of breast cancer risk factors in Isfahan university affiliated academical health centers. In this research, demographic, lifestyle, and fertility information was collected from 1001 people in Isfahan, Iran, 92 of whom tested positive for breast cancer. Obesity have been found to be positively correlated with multiple cancers including breast cancer, but we couldn't identify this factor as a risk factor in our study [29]. Out of all the factors that were evaluated in our study, only 2 factor reached statistical significance: consumption of alcohol and taller stature. Alcohol consumption and its role in breast cancer have been discussed previously in different studies [30, 31]. In a 2023 literature review, authors stated that the association of alcohol consumption with increased breast cancer incidence is mediated by alcohol‐induced renal burden and nontraumatic rhabdomyolysis, leading to hyperphosphatemia and phosphate toxicity and the World Health Organisation (WHO) stated that many people, including women, are not aware that breast cancer is the most common cancer caused by alcohol among women globally [32]. Global cancer incidence related to alcohol consumption was estimated from an analysis of GLOBOCAN 2020 data by WHO's International Agency for Research on Cancer (IARC) [33]. Cancer attributed to alcohol consumption accounted for 4.1% of all new cancer cases globally in 2020. At 10 g of alcohol per drink, daily alcohol consumption was classified as moderate (< 20 g), risky (20–60 g), and heavy (> 60 g). Heavy, risky, and moderate consumption of alcohol accounted for 46.7%, 39.4%, and 13.9% of new cancer cases, respectively. Lighter consumption of alcohol up to 10 g a day accounted for 41,300 new cancer cases. Furthermore, among potentially modifiable risk factors associated with the incidence of US cancer cases and deaths in 2014, alcohol intake ranked just behind cigarette smoking and excess body weight, accounting for 5.6% of cancer cases and 4% of cancer deaths [34]. Furthermore, there have been several studies, showing that height can be a risk factor for breast cancer. A 2015 study by Zhang et al. showed that the pooled relative risk of breast cancer was 1.17 per 10 cm increase in height. It should also be noted that, in our study 2 factors, taking vegetables (*p*‐value = 0.063) and history of benign breast cancer (*p* = 0.087) were marginally significant. A systematic review study with the title of “Fruits, vegetables and breast cancer risk: a systematic review and meta‐analysis of prospective studies” studied the abovementioned matter. The author concluded that high intake of fruits, and fruits and vegetables combined, but not vegetables, is associated with a weak reduction in risk of breast cancer [35]. The same results about vegetables were also reported in another review article which authors concluded a borderline inverse association between pre‐diagnostic intake of fruit and overall survival of breast cancer, whereas intake of vegetables was not associated with survival [36]. In a case‐control study in China, “History of benign breast disease and risk of breast cancer among women in China: a case–control study,” authors concluded that a personal history of BBD is associated with an increased risk of future breast cancer among women in China [37].

We also evaluated several other risk factors such as lifestyle factors (exercise, diets, fruits and vegetables, taking supplements, taking vitamin D…), demographic factors (Age, job, marital status, height, BMI) and reproductive factors (age of menstruation and menopause, history of other cancers, infertility). No significant relationship was found between the above‐mentioned factors and breast cancer. In a 2011 study, Saki et al. evaluated the risk factors of breast cancer in women aged less than 40 in Imam Khomeini hospital, Tehran, Iran. Their analysis showed that factors such as family history of breast cancer, family history of ovarian cancer, history of benign breast disease, low menarche age, irregular menstruation, lack of physical activity, and exposure to stressful events are effective factors in getting breast cancer [38]. A previous investigation involving 4820 women aged 35–70 years from Yazd, Iran, reported the following adjusted odds ratios for breast cancer: breastfeeding duration < 11 months (OR = 2.78), postmenopausal hormone therapy (OR = 2.07), menopause onset at age ≥ 46 years (OR = 1.71), history of abortion (OR = 1.63), oral contraceptive use (OR = 1.59), menarche at age ≤ 13 years (OR = 1.49), and overweight/obesity (OR = 1.45) [39].

Considering the high prevalence of breast cancer among the Irani women and its preventability, as well as the lack of a comprehensive and universal prevention and screening program, it seems that in addition to the actions of healthcare providers in the direction of promoting lifestyle education issues such as reducing Weight and exercise, etc., planning to integrate prevention and screening programs is also necessary. The most important one could be our relatively small sample size in the comparative analysis. A larger population from multicenter sources are required to investigate our results. Another limitation was not using standardized diet and physical active questionnaires due to a lack of adequate timing which should be considered in future studies.

### 4.1 Strengths and Limitations

This study possesses several notable strengths. Primarily, it addresses a critical gap in the literature by providing targeted insights into the risk factor profile for breast cancer specifically among middle‐aged women a demographic that is frequently underrepresented in epidemiological research, particularly in regions without organized screening programs. The cross‐sectional design, while inherently limited in its ability to infer causality, was appropriate for the exploratory aim of capturing a comprehensive snapshot of associations between a wide array of potential risk factors and breast cancer prevalence within this specific cohort. The systematic approach to data collection and analysis further strengthens the study, offering a clear and detailed overview of the observed trends that can generate hypotheses and inform the design of future, larger‐scale investigations.

However, the findings must be interpreted in light of several inherent limitations. First, the low incidence of breast cancer cases identified within the sample restricts the statistical power and generalizability of the findings, necessitating caution when extrapolating them to the broader population of middle‐aged women. Second, the cross‐sectional nature of the study precludes the establishment of causal or temporal relationships between the identified risk factors and breast cancer development. Third, the study population, recruited from specific health centers, may not fully represent the socioeconomic, ethnic, and geographic diversity of all middle‐aged women, thereby limiting the applicability of the results to other demographic groups or settings. Finally, despite efforts to collect data on a comprehensive set of variables, the potential influence of unmeasured or residual confounding factors cannot be entirely dismissed, and these may have impacted the observed associations.

## 5 Conclusion

This study examined risk factors associated with breast cancer in middle‐aged women and provided preliminary insights into these relationships. Given the low incidence observed in our sample, the findings are descriptive and exploratory, and should not be interpreted as representative of the broader breast cancer population. Nevertheless, these observations may help identify potential patterns within the studied cohort and inform future research with larger samples and population‐based designs. Studies with larger sample sizes are recommended to evaluate the sensitivity and specificity of diagnostic tools as well as to more accurately identify risk factors.

## Author Contributions

**Hourieh Ansari:** project administration, software, methodology, validation, writing – original draft. **Sara Yadollahi:** methodology, data curation, writing – review and editing, writing – original draft. **Soheila Allahyari:** writing – review and editing, investigation. **Ali Hajigholami:** conceptualization, methodology, funding acquisition, formal analysis, writing – review and editing, writing – original draft, supervision, visualization.

## Funding

The authors have nothing to report.

## Disclosure

The lead author Ali Hajigholami affirms that this manuscript is an honest, accurate, and transparent account of the study being reported; that no important aspects of the study have been omitted; and that any discrepancies from the study as planned (and, if relevant, registered) have been explained.

## Ethics Statement

This study was performed in line with the principles of the Declaration of Helsinki and was approved by the Ethics Committee of Isfahan University of Medical Sciences (IR.MUI.MED.REC.1401.186). The studies were conducted in accordance with the local legislation and institutional requirements. Written informed consent for participation in this study was provided by the participants or their legal guardians/next of kin.

## Conflicts of Interest

The authors declare that the research was conducted in the absence of any commercial or financial relationships that could be construed as a potential conflict of interest.

## Data Availability

The data that support the findings of this study are available from the corresponding author upon reasonable request.
